# An epigenetic GPI anchor defect impairs TLR4 signaling in the B cell transdifferentiation model for primary human monocytes BLaER1

**DOI:** 10.1038/s41598-021-94386-z

**Published:** 2021-07-22

**Authors:** Julia Wegner, Thomas Zillinger, Thais Marina Schlee-Guimaraes, Eva Bartok, Martin Schlee

**Affiliations:** 1 Institute of Clinical Chemistry and Clinical Pharmacology, University Hospital Bonn, Bonn, Germany; 2 Unit of Experimental Immunology, Department of Biomedical Sciences, Institute of Tropical Medicine, Antwerp, Belgium; 3 Present Address: Institute of Immunology, Philipps-University Marburg, Marburg, Germany

**Keywords:** Toll-like receptors, Monocytes and macrophages, Immunological models, Gene silencing, Inflammasome

## Abstract

Antigen-presenting myeloid cells like monocytes detect invading pathogens via pattern recognition receptors (PRRs) and initiate adaptive and innate immune responses. As analysis of PRR signaling in primary human monocytes is hampered by their restricted expandability, human monocyte models like THP-1 cells are commonly used for loss-of-function studies, such as with CRISPR-Cas9 editing. A recently developed transdifferentiation cell culture system, BLaER1, enables lineage conversion from malignant B cells to monocytes and was found superior to THP-1 in mimicking PRR signaling, thus being the first model allowing TLR4 and inflammasome pathway analysis. Here, we identified an important caveat when investigating TLR4-driven signaling in BLaER1 cells. We show that this model contains glycosylphosphatidylinositol (GPI) anchor-deficient cells, which lack CD14 surface expression when differentiated to monocytes, resulting in diminished LPS/TLR4 but not TLR7/TLR8 responsiveness. This GPI anchor defect is caused by epigenetic silencing of *PIGH*, leading to a random distribution of intact and *PIGH*-deficient clones after single-cell cloning. Overexpressing PIGH restored GPI-anchored protein (including CD14) expression and LPS responsiveness. When studying CD14- or other GPI-anchored protein-dependent pathways, researchers should consider this anomaly and ensure equal GPI-anchored protein expression when comparing cells that have undergone single-cell cloning, e. g. after CRISPR-Cas9 editing.

## Introduction

Monocytes and monocyte-derived cells are an essential part of the human innate immune system. They express a variety of pattern recognition receptors (PRRs) that can sense conserved components of microbes, known as pathogen-associated molecular patterns (PAMPs). To eliminate potential pathogens, signaling cascades are initiated upon PRR activation that can lead to the production of type I interferon, proinflammatory cytokines such as interleukin 6 (IL-6) and pro-IL-1β as well as the activation of the inflammasome, a multiprotein complex that proteolytically activates IL-1β and induces pyroptotic cell death. PRRs can be classified into several families, including C-type lectin receptors (CLRs), NOD receptors, NOD-like receptors (NLRs), Toll-like receptors (TLRs), cytosolic DNA receptors, and RIG-I-like receptors (RLRs)^1^. While CLRs and NODs are found on the cell surface, TLRs are located either on the plasma membrane (e. g. TLR4) or the endosomal membrane (e. g. TLR8). In contrast, NLRs, the DNA receptors cGAS and AIM2, and RLRs can be found in the cytosol of the cell^1,2^.

Immortalized myeloid cell lines such as THP-1 and U-937 have been widely used to investigate innate immune pathways in the human system. However, PRR signaling in these cells can differ dramatically from their physiological counterparts. One important deviation is in TLR4 signaling. Compared to primary human monocytes, U-937 cells demonstrate prominent transcriptional differences to LPS-mediated TLR4 activation^3^. Moreover, THP-1 cells are far less responsive to LPS in the first place as they express only minimal amounts of the TLR4 co-receptor CD14^4^.

In 2013, a new sophisticated model for monocytes was introduced—a human transdifferentiation cell culture system, known as BLaER1^5^. These cells were derived by transducing the B cell-precursor leukemia RCH-ACV cell line with a construct of the myeloid transcription factor C/EBPα fused to the ligand-binding domain of the estrogen receptor (C/EBPα-ER-IRES-GFP). Treatment with β-estradiol, IL-3, and M-CSF causes BLaER1 cells to transdifferentiate from a highly proliferative B cell state into a non-proliferative, monocytic one. The transcriptome of BLaER1 monocytes closely resembles that of primary human monocytes, and also many of the PRR signaling pathways are highly similar between these cells, including TLR4 and inflammasome signaling^6^. Thus, the BLaER1 B cell transdifferentiation system is currently the only human monocyte model that enables the analysis of the TLR4 and inflammasome signaling pathways. Alternative inflammasome activation, downstream of TLR4 activation, takes place in primary human monocytes and transdifferentiated BLaER1 but is not functional in THP-1 cells and murine macrophages. CRISPR-Cas9-mediated gene knock-outs (KOs) in the BLaER1 model allowed the identification of proteins involved in this signaling entity^7^.

Virtually all studies to date using CRISPR-Cas9 genome editing in BLaER1, THP-1, and U-937 cells have utilized limiting-dilution seeding for the generation of monoclonal cell lines. CRISPR-Cas9-mediated gene editing via non-homologous end joining induces spontaneous insertions or deletions (indels) in the targeted gene. Thus, this approach generally results in a polyclonal cell population in which every individual cell (and allele) can harbor different indels. In BLaER1 cells, this process can be performed in their B cell state, in which a high proliferation rate facilitates the generation of monoclonal cell lines harboring specific indels.

In this study, we report a defect in TLR4 signaling in a subpopulation of BLaER1 cells. The isolation and expansion of single cells results in monoclonal BLaER1 lines, with some of them demonstrating attenuated LPS responsiveness. We show that this phenotype originates from epigenetic silencing of PIGH, a protein involved in GPI anchor biosynthesis, which is necessary for CD14 surface expression on transdifferentiated BLaER1 cells. While the BLaER1 population contains a mixture of PIGH-expressing and -deficient cells, limiting-dilution seeding can result in monoclonal cell lines with dramatically different TLR4 signaling phenotypes. As LPS priming is common in the field of inflammasome research, where BLaER1 cells have become widely used, the use of a PIGH-deficient cell line causing diminished LPS responsiveness could lead to unreliable results. Therefore, our study provides an important caveat for the use of BLaER1 cells in investigating TLR4-driven pathways.

## Results

### A subpopulation of BLaER1 monocytes exhibits TLR4 signaling deficiency and lack of CD14 surface expression

**Figure 1:**
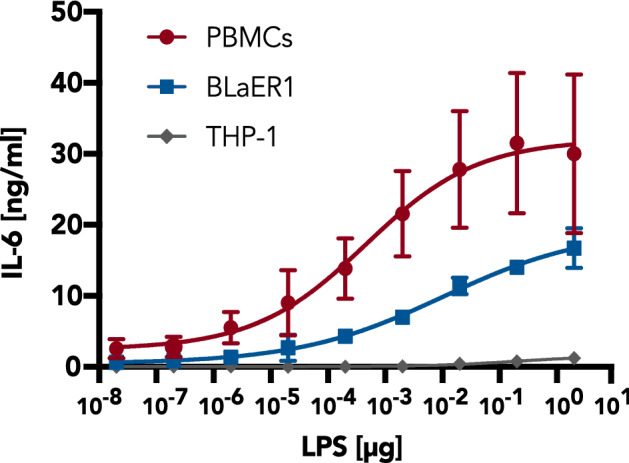
BLaER1 but not THP-1 monocytes model the LPS response of human PBMCs. After stimulation of human PBMCs, BLaER1 monocytes, and THP-1 monocytes for 16 h with LPS, IL-6 in the supernatant was measured by ELISA. Values correspond to the mean ± SD of n = 2 independent experiments (for BLaER1 and THP-1) or n = 5 donors (for PBMCs).

**Figure 2:**
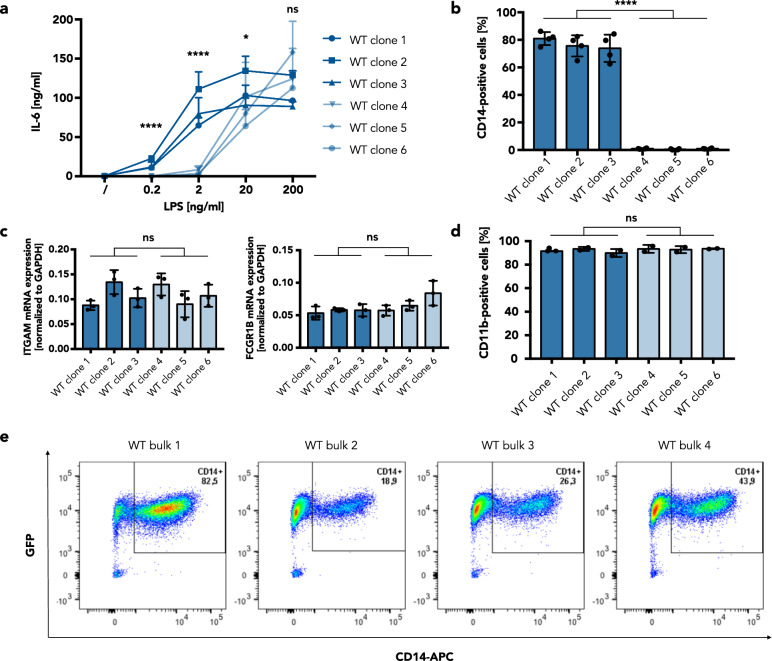
Reduced LPS responsiveness in BLaER1 monocytes is caused by a lack of CD14 surface expression. (**a**) After stimulation of transdifferentiated BLaER1 monoclonal cell lines for 16 h with LPS, IL-6 in the supernatant was measured by ELISA. Values correspond to the mean + SD of n = 3 independent experiments. (**b**) Percentage of cells expressing CD14 on the cell surface among all GFP-positive cells, assessed by FACS analysis of transdifferentiated BLaER1 clones. Shown is the mean ± SD of n = 4 independent measurements. Individual values are visualized as dots. (**c**) mRNA expression of the monocyte/macrophage markers *ITGAM* and *FCGR1B* in transdifferentiated WT clones, measured by qRT-PCR. Individual values are shown as dots. Columns correspond to the mean ± SD of n = 3 independent measurements, normalized to *GAPDH* expression. (**d**) Percentage of BLaER1 cells expressing CD11b on the cell surface among all GFP-positive cells after transdifferentiation for 5 days. Columns show the mean ± SD of n = 2 independent measurements. Dots represent the individual values. (**a–d**) Monoclonal cell lines with higher responsiveness to LPS (dark blue) were compared to cell lines with lower responsiveness to LPS (light blue) by Mann–Whitney test (ns—not significant, *p ≤ 0.05, ****p ≤ 0.0001). (**e**) FACS analysis of bulk CD14 surface expression in BLaER1 cells transdifferentiated for 5 days. The percentage of cells contained in the GFP+/CD14+ population is labeled in the upper right corner of each plot.

The comparison of the LPS/TLR4 response of primary human peripheral blood mononuclear cells (PBMCs) with THP-1 and transdifferentiated BLaER1 cells revealed that BLaER1 monocytes but not THP-1 cells can elicit a proper IL-6 response to LPS (Fig. 1). However, while generating monoclonal KO BLaER1 cell lines, we observed that many of the lines demonstrated a reduced LPS/TLR4 response (data not shown), although the genes targeted for KO were not known to be involved in TLR4 signaling. Furthermore, the defect in the LPS response could not be restored by reconstitution of the targeted genes (data not shown). To address whether this phenotype was already present in the parental cells or if it resulted from steps within the genome editing procedure, monoclonal cell lines of wildtype (WT) BLaER1 B cells were generated by limiting-dilution seeding, in the same way as it has been done for the KO cell lines. Transdifferentiation and stimulation of these WT clones with LPS led to diminished IL-6 and TNFα production in several of the WT lines (clones 4–6), similar to what was detected for the KO cell lines before, clearly demonstrating that the TLR4 signaling deficiency observed was independent of both electroporation and CRISPR-Cas9 genome editing (Fig. 2a, Supplementary Fig. S1). Notably, cytokine production in these clones was only reduced for LPS concentrations of 2 ng/ml and below, whereas no difference or even an enhancement was detectable at a higher concentration of 200 ng/ml (Fig. 2a, Supplementary Fig. S1). LPS is sensed by a complex of TLR4 and myeloid differentiation factor 2 (MD-2)^8^. To investigate whether downregulation of these factors is involved in the observed diminished LPS responsiveness of WT clones 4–6, we quantified *TLR4* and *MD-2* mRNA levels. However, no differential expression to WT clones 1–3 could be observed (Supplementary Fig. S2). Because the TLR4 accessory protein CD14 has been shown to be necessary for proinflammatory cytokine production at low LPS concentrations^9,10^, we then measured CD14 surface expression on each of the clones by flow cytometry. Strikingly, in the WT clones with diminished LPS responsiveness (4–6), CD14 was completely absent in the monocyte state whereas the TLR4-signaling-competent clones (1–3) showed surface expression of CD14 (Fig. 2b, Supplementary Fig. S3).

As CD14 is a classical monocyte marker^11^ and has also been described as a marker of transdifferentiation in the BLaER1 model^5^, we speculated that the transdifferentiation to monocytes/macrophages was disturbed in these clones, which would explain the lack of CD14 expression. We therefore performed qRT-PCR for the macrophage markers *ITGAM* and *FCGR1B* as well as surface staining for the macrophage marker CD11b to monitor the transdifferentiation process^5^. However, no significant differences in these markers of transdifferentiation could be observed between the CD14-positive and CD14-negative clones (Fig. 2c,d).

To investigate whether the downregulation of CD14 in these clones occurred prior to limiting-dilution seeding, we analyzed CD14 surface expression in transdifferentiated polyclonal WT populations obtained from different frozen BLaER1 stocks. Strikingly, the percentage of CD14-positive cells varied from 18.9 to 82.5% (Fig. 2e), indicating a clonal effect that was not induced by cell separation but already present in the bulk population.

### Diminished CD14 expression goes along with a general defect in GPI-anchored protein expression

**Figure 3:**
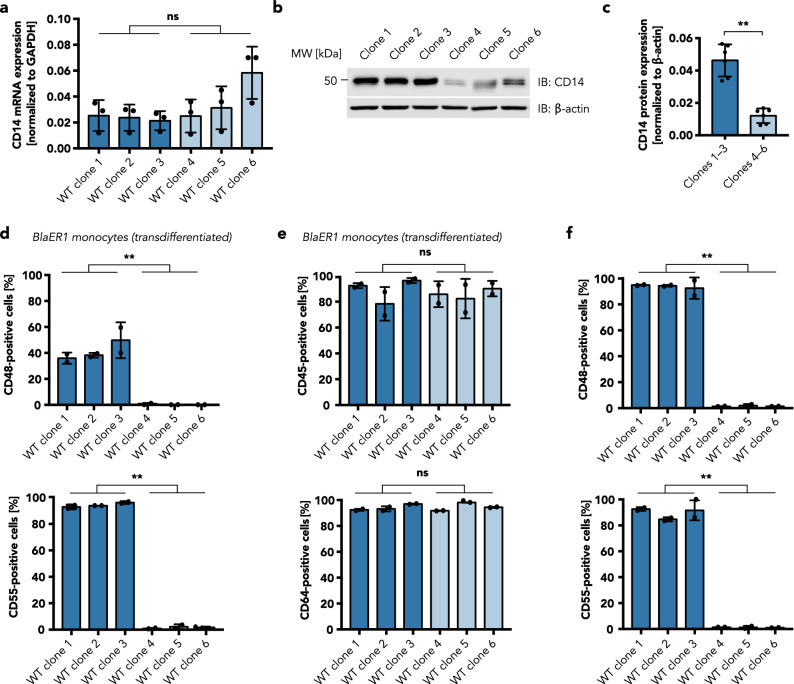
Loss of CD14 on the cell surface in BLaER1 monocytes is the result of a general GPI anchor defect. (**a**) *CD14* mRNA expression of BLaER1 clones, quantified by qRT-PCR. Shown is the mean ± SD of n = 3 independent measurements, normalized to *GAPDH* expression. Dots represent individual values. (**b**) Western blot analysis of CD14 expression. One representative blot of two is shown. Full-length blots are presented in Supplementary Figure S9. (**c**) Quantification of the CD14 protein in (**b**). CD14 expression was normalized to the housekeeping protein β-actin. The mean ± SD of pooled data from three different monoclonal cell lines from n = 2 independent measurements is shown. Dots represent the individual values. (**d–f**) Surface expression of the GPI-anchored proteins CD48 and CD55 (**d,f**) and the transmembrane proteins CD45 and CD64 (**e**) was measured by flow cytometry in transdifferentiated (**d,e**) or undifferentiated (**f**) BLaER1 monoclonal cell lines. Shown is the percentage of cells expressing the protein of interest among all GFP-positive cells from n = 2 independent measurements (mean ± SD). Individual values are depicted as dots. (**a,c–f**) Monoclonal cell lines with higher responsiveness to LPS (dark blue) were compared to cell lines with lower responsiveness to LPS (light blue) by Mann–Whitney test (ns—not significant, **p ≤ 0.01).

To characterize the mechanism of the loss of CD14 surface expression, we analyzed both CD14 total protein and mRNA expression. No reduction in *CD14* mRNA levels could be detected for clones with diminished LPS responsiveness, arguing for a post-transcriptional cause of CD14 loss (Fig. 3a). Moreover, intracellular CD14 was readily detectable in all clones, including those demonstrating a defect in TLR4 signaling (Fig. 3b): Western blot analysis revealed an average reduction of total protein expression of only 3.8-fold (Fig. 3c), suggesting that it may be instead a post-translational modification of CD14 or change in its transport to the plasma membrane that is causing this effect.

Normally, CD14 is tethered to the plasma membrane by a glycosylphosphatidylinositol (GPI) anchor that is covalently attached to the C terminus of the protein. In humans, more than 150 GPI-anchored proteins with diverse cellular functions are known^12^. To investigate whether the observed defect is specific for CD14, we analyzed the surface expression of two other GPI-anchored proteins, CD48 and CD55, as well as that of the transmembrane proteins CD36, CD45, CD64, CD163, and CD191 after transdifferentiation of BLaER1 monoclonal cell lines by flow cytometry. Similar to CD14, the surface expression of CD48 and CD55 was found to be completely absent in the clones with reduced LPS responsiveness (Fig. 3d, Supplementary Fig. S3). In contrast, the expression of all of the analyzed transmembrane proteins was unchanged, pointing to a specific defect in GPI-anchored protein expression (Fig. 3e, Supplementary Fig. S4).

Although defects in GPI anchor biosynthesis have been shown to be embryonically lethal in mice^13^, we could not observe a reduced metabolic activity of GPI-deficient clones (4–6) compared to GPI-positive cells (clones 1–3), demonstrating that the lack of GPI anchorage does not affect cellular viability in the BLaER1 model (Supplementary Fig. S5).

While CD14 is a monocyte marker and only expressed after transdifferentiation of BLaER1 cells, CD48 and CD55 can also be found in the B cell state of the cell line. Flow cytometric analysis of undifferentiated BLaER1 cells confirmed the complete absence of these proteins on the cell surface of the clones with defective LPS responsiveness (4–6; Fig. 3f). This result emphasizes that the observed GPI anchor defect is inherent to a subpopulation of the cell line and not induced by the transdifferentiation process.

### The GPI anchor defect is caused by a lack of PIGH expression

**Figure 4:**
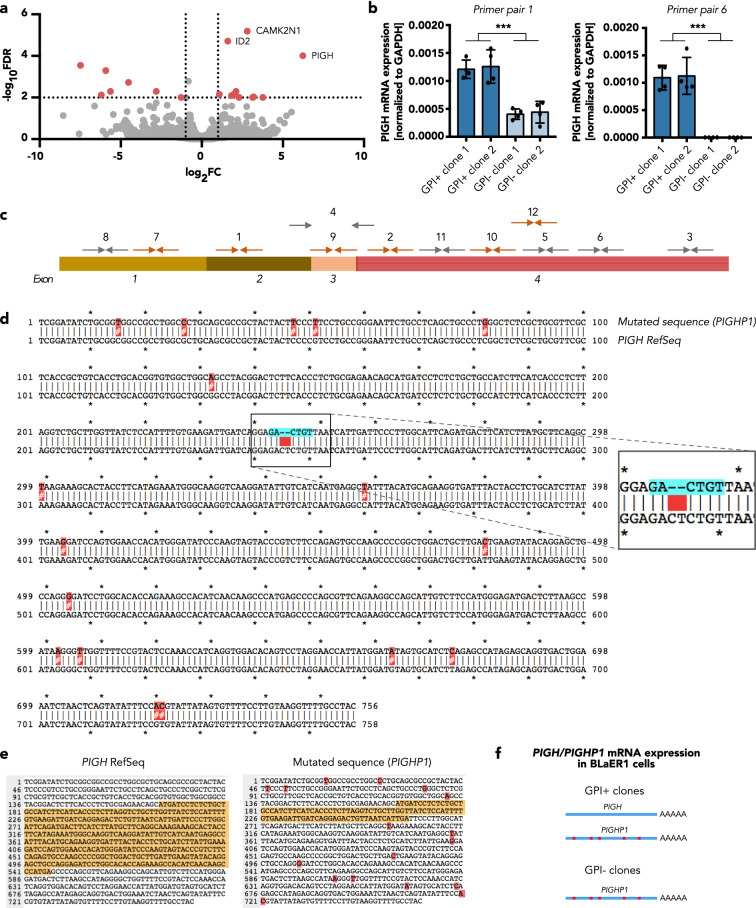
Absence of functional PIGH expression in BLaER1 clones with reduced LPS responsiveness. (**a**) Volcano plot of 3′ mRNA sequencing from undifferentiated BLaER1 cells, showing differentially expressed genes between GPI-positive (1–3) and GPI-negative (4–6) monoclonal cell lines. Genes with a -log_10_ false discovery rate (FDR) > 2 and log_2_ fold change (FC) > 1 (downregulated in GPI-negative clones) or < -1 (upregulated in GPI-negative clones) are shown as red dots. (**b**) *PIGH* mRNA expression of GPI-positive (GPI +) and -negative (GPI-) clones was quantified by qRT-PCR using two different primer pairs (1 and 6) and normalized to *GAPDH* mRNA expression. GPI + and GPI- clones correspond to the WT clones depicted in Figs. 2 and 3 as follows: GPI + clone 1 = WT clone 1, GPI + clone 2 = WT clone 2, GPI- clone 1 = WT clone 4, and GPI- clone 2 = WT clone 6. The mean ± SD of data from n = 4 independent measurements is shown. Individual values are visualized as dots. Statistical significance was determined using Mann–Whitney test (***p ≤ 0.001). (**c**) Schematic visualization of *PIGH* mRNA. Shown is a summary of the results obtained from qRT-PCR with multiple primer pairs (arrows with numbers). Gray arrows indicate that no (or only minor) signals were obtained for *PIGH* mRNA expression of the GPI-negative clones, whereas substantial amplification was achieved with the primers depicted as orange arrows. (**d**) Alignment of the *PIGH* mRNA Reference Sequence (RefSeq) with the sequence obtained from GPI-negative clones by Sanger sequencing, which corresponds to *PIGHP1*. Point mutations are highlighted in red. The location of a two-nucleotide deletion is highlighted in blue. (**e**) Open reading frame (ORF) prediction for the *PIGH* RefSeq and the mutated sequence of the pseudogene *PIGHP1*. The complete predicted ORF is highlighted in yellow. The two-nucleotide deletion in *PIGHP1* leads to a frameshift that causes a truncated ORF. (**f**) Schematic representation of *PIGH* mRNA expression in GPI-positive (GPI +) and -negative (GPI-) BLaER1 monoclonal cell lines. While GPI-positive clones express both the *PIGH* and the *PIGHP1* mRNA, only the *PIGHP1* mRNA can be detected in the GPI-negative clones.

GPI anchor biosynthesis is a complex multi-step process that takes place at the endoplasmic reticulum and involves a multitude of enzymes^12^. To identify the cause of the GPI anchor defect in BLaER1 cells, we performed a 3′ mRNA sequencing (3′ mRNA-seq) of undifferentiated BLaER1 cells from GPI-positive and -negative clones. The most significantly downregulated mRNA in the GPI-negative clones was found to be *phosphatidylinositol N-acetylglucosaminyltransferase subunit H* (*PIGH*), which is involved in the first step of GPI anchor biosynthesis (Fig. 4a). Together with six other proteins (PIGA, PIGC, PIGP, PIGQ, PIGY, and DPM2), PIGH forms the GPI-GlcNAc transferase complex that transfers *N*-acetylglucosamine (GlcNAc) from UDP-GlcNAc to the phosphatidylinositol (PI) lipid^12^. To confirm the downregulation of *PIGH* mRNA expression in GPI-negative BLaER1 clones, qRT-PCR using gene-specific primers was performed. Strikingly, varying results were obtained with primers binding to different regions of the *PIGH* mRNA: While some of the assessed primer pairs showed a complete absence of *PIGH* mRNA in GPI-negative clones (primer pairs 3–6, 8, and 11; Fig. 4b,c, Supplementary Fig. S6), others were still capable of amplifying the sequence (primer pairs 1, 2, 7, 9, 10, and 12; Fig. 4b,c, Supplementary Fig. S6). This discrepancy could either suggest that specific regions of the *PIGH* mRNA are still expressed in GPI-deficient clones, or that a *PIGH*-related transcript is amplified with these primer pairs. We found that primers binding to the 3′ end of the *PIGH* mRNA (primer pairs 3, 5, and 6) could not amplify the sequence in the GPI-negative BLaER1 cells, thus corroborating the results from the 3′ mRNA-seq and clearly demonstrating that the 3′ end of the *PIGH* mRNA is lost.

To further investigate the partial loss of *PIGH* mRNA, cDNA from GPI-positive and -negative clones was amplified by PCR (forward primer of pair 7, reverse primer of pair 12), and the PCR products were then analyzed by Sanger sequencing. For both of the GPI-negative clones analyzed, clear signals with no overlapping peaks were obtained, indicative of a single genotype (Supplementary Fig. S7). Alignment of the sequence obtained from GPI-negative clones with the *PIGH* NCBI Reference Sequence (RefSeq) revealed the presence of 17 point mutations and a two-nucleotide deletion within the analyzed region (Fig. 4d). Some of these mutations are located at the immediate 3′ end of the qRT-PCR primer binding sites and therefore disturb the amplification by PCR, which explains the absence of a PCR product for GPI-negative clones in these cases (primer pairs 8 and 11). Sequence comparison using NCBI Basic Local Alignment Search Tool (BLAST) revealed that the mutated sequence matches the mRNA transcribed from a *PIGH* pseudogene, *PIGH pseudogene 1 (PIGHP1)*, indicating that GPI-negative clones express *PIGHP1* but not *PIGH*. An open reading frame (ORF) prediction showed that the frameshift mutation induced by the deletion of two nucleotides in *PIGHP1* results in a truncated ORF that is unlikely to produce a functional protein (Fig. 4e).

In contrast, the result for the GPI-positive clones showed superimposed signals, which suggests the presence of two different sequences (Supplementary Fig. S7). Peak parsing and deconvolution revealed that one of the sequences matched *PIGHP1* from the GPI-negative clones, whereas the second sequence corresponded to the *PIGH* RefSeq.

In summary, sequence analysis revealed that GPI-negative BLaER1 cells solely express the *PIGHP1* mRNA that is unable to produce a functional protein, whereas in GPI-positive clones both *PIGH* and *PIGHP1* are transcribed (Fig. 4f).

### Loss of PIGH mRNA expression originates from epigenetic downregulation of the gene

**Figure 5:**
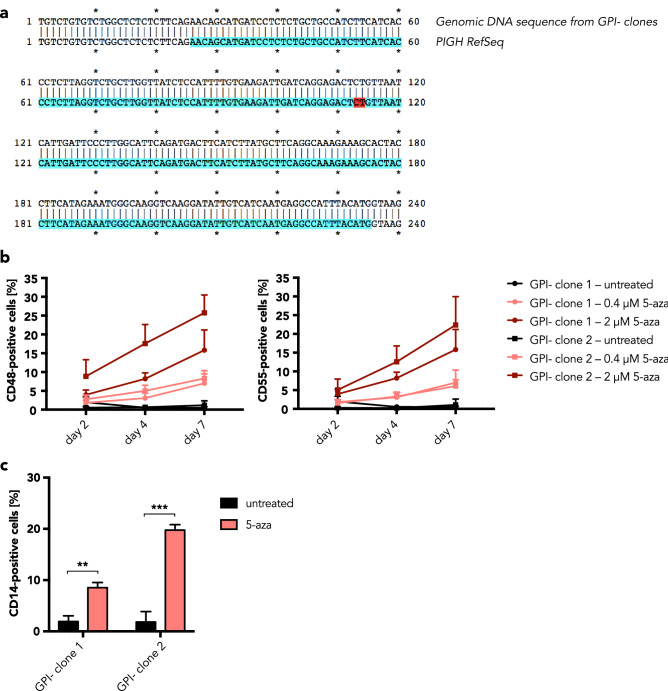
*PIGH* is epigenetically downregulated in GPI-negative BLaER1 cells. (**a**) Alignment of the *PIGH* locus RefSeq with the Sanger sequencing result for genomic DNA from the GPI-negative clones. Exon 2 is depicted in blue. The two nucleotides that are deleted in the *PIGHP1* sequence are shown in red. (**b**) Two GPI-negative BLaER1 monoclonal cell lines (1, circle and 2, square) were treated with 0.4 µM (light red) or 2.0 µM (dark red) 5-aza-2′-deoxycytidine (5-aza) or left untreated (black) for 2, 4, and 7 days. The percentage of CD48- or CD55-positive cells among GFP-expressing cells was determined by FACS analysis. Shown is the mean + SD of data from n = 3 independent experiments. (**c**) After treatment of GPI-negative BLaER1 monoclonal cell lines with 0.4 µM 5-aza or leaving them untreated for 4 days, cells were transdifferentiated for 5 days. The percentage of CD14-positive cells among GFP-expressing cells was determined by FACS analysis. Shown is the mean + SD of data from n = 3 independent experiments. 5-aza-treated cells were compared to untreated control cells using unpaired t tests (**p ≤ 0.01, ***p ≤ 0.001).

We then determined why the *PIGH* mRNA is lost in GPI-negative clones. Here, two scenarios were possible: Either these cells have two deleted *PIGH* genes or they have the same genotype as the GPI-positive clones but *PIGH* is epigenetically silenced. Thus, we analyzed the genomic DNA in the region containing the two-nucleotide deletion using PCR and Sanger sequencing. Genotyping revealed the presence of the *PIGH* sequence in both GPI-positive and -negative cells, arguing for epigenetic silencing of the *PIGH* gene in the GPI-negative cells (Fig. 5a).

To confirm the involvement of an epigenetic mechanism in the downregulation of *PIGH* mRNA expression and the resulting GPI anchor biosynthesis defect in GPI-negative BLaER1 cells, we tested whether the demethylating drug 5-aza-2′-deoxycytidine (5-aza) could restore GPI-anchored protein expression. Over the course of 7 days, a gradual increase in the percentage of CD48- and CD55-positive cells was detected in samples treated with 5-aza (Fig. 5b). Furthermore, 5-aza treatment for 4 days before transdifferentiation of GPI-negative BLaER1 clones partially restored CD14 surface expression (Fig. 5c). These data demonstrate that promoter methylation is responsible for the loss of GPI anchor expression in GPI-negative BLaER1 cells.

### Re-expression of functional PIGH rescues TLR4 signaling but does not affect other PRRs

**Figure 6:**
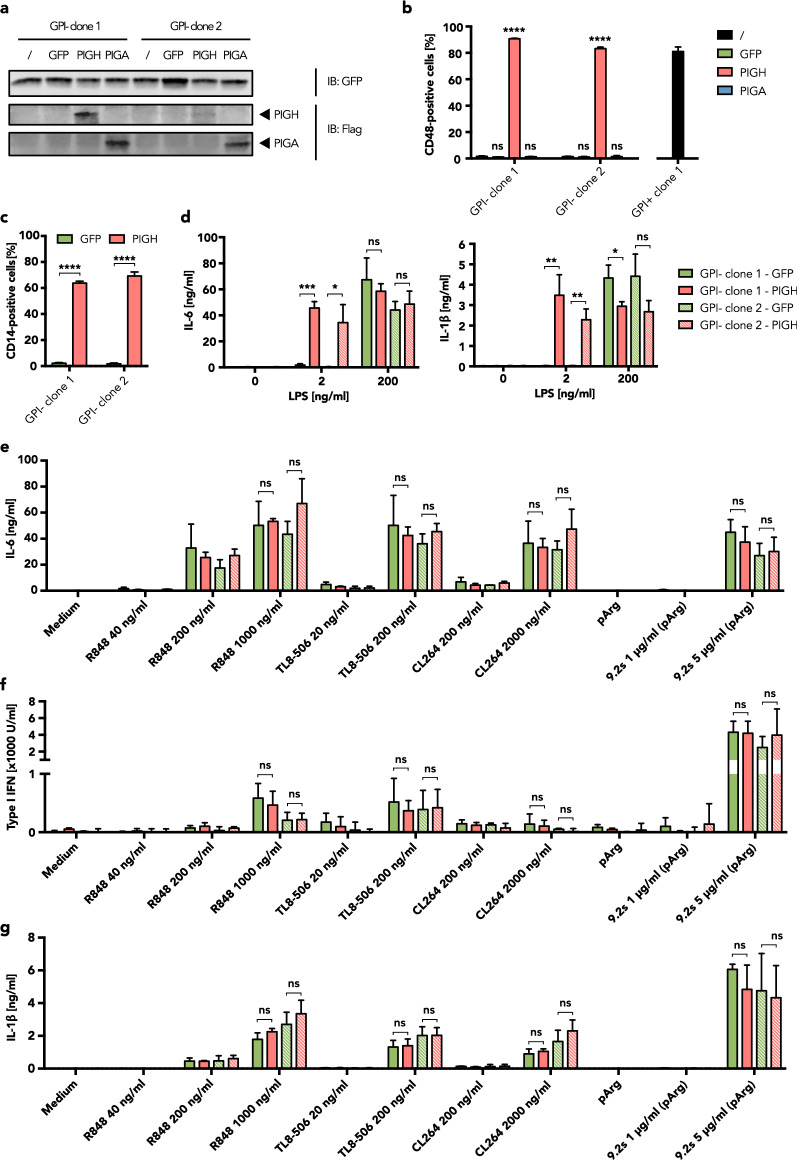
Rescue of GPI-anchored protein expression and TLR4 signaling by PIGH re-expression. (**a**) Western blot analysis of GPI-negative (GPI-) BLaER1 clones that were either transduced with a lentivirus encoding for GFP, PIGH-Flag, or PIGA-Flag, or not transduced (/) as a negative control. One representative blot of three is shown. Full-length blots are presented in Supplementary Figure S9. (**b**) GPI-negative BLaER1 cells stably expressing GFP, PIGH-Flag, or PIGA-Flag were analyzed by flow cytometry. The percentage of CD48-expressing cells among GFP-positive cells is shown as the mean + SD from n = 3 independent measurements. For comparison, CD48 surface expression of a GPI-positive (GPI +) BLaER1 monoclonal cell line is shown on the right-hand side. Two-way ANOVA followed by Dunnett’s multiple comparisons test was used to determine statistical significance. Results from comparing cells transduced with either GFP, PIGH-Flag, or PIGA-Flag with the negative control (not transduced, /) are shown (ns – not significant, ****p ≤ 0.0001). (**c**) GPI-negative BLaER1 cells stably expressing GFP or PIGH-Flag were transdifferentiated for 5 days and then analyzed by flow cytometry. The percentage of CD14-expressing cells among GFP-positive cells is shown as the mean + SD from n = 3 independent measurements. (**d**) IL-6 and IL-1β in the supernatant were determined by ELISA 16 h after the stimulation of BLaER1 monoclonal cell lines, stably expressing GFP or PIGH-Flag, with LPS or medium control (0). Values correspond to the mean + SD of n = 3 independent experiments. (**e–g**) BLaER1 clones stably expressing GFP or PIGH-Flag were stimulated for 16 h with four different TLR7/TLR8 ligands and the supernatant was used to determine IL-6 (**e**) and IL-1β (**g**) concentrations by ELISA, or type I IFN concentration by HEK blue assay (**f**). Data for stimulation with R848 40 ng/ml and 200 ng/ml, TL8-506 20 ng/ml, and CL264 200 ng/ml are depicted as mean + SD of n = 2 independent experiments. For all other stimuli and concentrations, the mean + SD of n = 3 independent experiments is shown. (**c–g**) BLaER1 cells stably expressing PIGH-Flag were compared to GFP-expressing control cells using unpaired t tests. Statistical significance is depicted as follows: ns—not significant, *p ≤ 0.05, **p ≤ 0.01, ***p ≤ 0.001, ****p ≤ 0.0001.

To confirm that *PIGH* downregulation is the cause of the observed TLR4 signaling defect in BLaER1 cells, we restored PIGH function in GPI-negative BLaER1 cells by overexpressing the WT protein using a lentiviral vector (Fig. 6a). Functional PIGH expression was found to eliminate the GPI anchor defect, and surface expression of CD48 and CD55 was completely rescued, whereas overexpression of GFP or PIGA had no such effect (Fig. 6b, Supplementary Fig. S8). Importantly, PIGH expression also restored CD14 surface expression in transdifferentiated GPI-negative BLaER1 clones (Fig. 6c). We further tested the TLR4 signaling capacity of cells overexpressing PIGH compared to GFP. The LPS responsiveness of the GPI-negative clones was completely restored by functional PIGH expression (Fig. 6d). These data demonstrate that the loss of the *PIGH* mRNA is solely responsible for the observed GPI anchor defect and reduced TLR4 signaling capacity of BLaER1 cells.

As CD14 has been described to be involved in TLR3, TLR7, and TLR9 signaling^14,15^, we also investigated the effect of PIGH reconstitution on the responsiveness of the cells to other PRR ligands. IL-6 and type I IFN production were measured after stimulation with the TLR7 ligand CL264, the TLR8-specific ligand TL8-506 as well as with the TLR7/8 ligands R848 and 9.2s RNA. In disparity with previous studies in HEK293T and murine cells^14^, we found that TLR7/8 sensitivity to small molecule ligands or short RNA was not influenced by GPI-anchored protein expression in BLaER1 monocytes (Fig. 6e,f). TLR7- and TLR8-induced inflammasome activation, as measured by IL-1β secretion, was unchanged after PIGH re-expression as well (Fig. 6g). We could also not detect any influence of GPI anchor expression on the cytokine production induced by activation of TLR2/TLR1 with Pam3CSK4 (Supplementary Fig. S8). Furthermore, the responsiveness of BLaER1 monocytes to ligands of the cytosolic nucleic acid receptors RIG-I and cGAS was not affected by PIGH re-expression (Supplementary Fig. S8). In summary, these data demonstrate that functional PIGH is essential for CD14-mediated, high affinity TLR4 signaling. However, neither CD14 nor any other GPI-anchored protein seem to be involved in proinflammatory cytokine and type I IFN production induced by TLR7, TLR8, TLR2/1, RIG-I, and cGAS activation in BLaER1 monocytes.

## Discussion

Transdifferentiated BLaER1 monocytes have become a widely used model cell line in the field of innate immunity owing to their ability to faithfully recapitulate many of the signaling pathways in primary monocytes^6^. However, our study reveals a major caveat of the use of BLaER1 monocytes for studying the TLR4 pathway and inflammasome activation that needs to be addressed. We have shown that BLaER1 cells harbor a GPI anchor defect that affects their responsiveness to LPS and is induced by epigenetic silencing of PIGH expression.

Since the original publication describing the establishment of the BLaER1 cell line^5^, an increasing number of studies have utilized BLaER1 monocytes, predominantly for the examination of inflammasome activation pathways. In most of these publications, CRISPR-Cas9 genome editing followed by single-cell isolation and expansion has been performed^6,7,16^. As shown here, this procedure results in GPI-positive and GPI-negative monoclonal cell lines as a random, KO-independent event. GPI-negative KO clones differ from the WT population not only in the specific CRISPR-Cas9-mediated depletion of gene expression but also lack GPI-anchored proteins on their surface. Such proteins include the monocyte marker and TLR4 co-receptor CD14, whose deficiency results in a diminished responsiveness of these cells to treatment with LPS. Using these clones for the investigation of inflammasome activation bears the risk of generating misleading data, because LPS stimulation, commonly used for inflammasome priming, would lead to diminished pro-IL-1β induction compared to the WT cells, independent of their genotype.

Thus, when working with BLaER1 cells, determining that the GPI-anchored protein expression is equal in each of the WT and KO cell lines is indispensable for achieving results that are solely determined by the specific genotype that was introduced. Alternatively, since in this study we showed that lentiviral expression of functional PIGH completely and permanently restores GPI-anchored protein expression in BLaER1 cells, the use of a PIGH-transduced BLaER1 cell line for CRISPR-Cas9 genome editing and subsequent monoclonal cell line generation could avoid the need for elaborate quantification of GPI anchor expression in the individual clones.

In addition to its role in TLR4 signaling, CD14 has been reported to enhance TLR7 and TLR9 signaling and to interact with TLR8^14^. However, we could not detect any prominent differences in the type I IFN and proinflammatory cytokine production induced by the TLR7/8 ligands R848 and 9.2s RNA, the TLR7-specific ligand CL264, or by the TLR8-specific ligand TL8-506 in GPI-negative compared to PIGH-transduced GPI-positive BLaER1 monocytes. This finding suggests that CD14 may have a cell-type- or species-specific function in TLR7/8 signaling and underlines the importance of BLaER1 monocytes as human monocyte model. Furthermore, we could not detect any impairment of the TLR2/1, RIG-I, and cGAS activation pathways in GPI-negative cells. Nonetheless, it cannot be ruled out that GPI-anchored proteins contribute to the cytokine production induced by the activation of other PRRs that have not been analyzed here or other basic physiological functions of human monocytes.

In a recent study, the BLaER1 model was used to investigate the role of genome organization by topologically associating domains (TADs) in inflammatory responses^17^. Given that depletion of the TAD-organizing protein CTCF in this study did not influence cell transdifferentiation but strongly diminished the cytokine response to LPS, it is tempting to speculate that an impairment of CTCF function might contribute to *PIGH* silencing in GPI-negative clones. Although our 3′ mRNA-seq analysis did not show a statistically significant downregulation of CTCF, a functional restriction cannot be excluded.

BLaER1 is not the first cell line described to harbor a defect in GPI anchoring. A previous study demonstrated that a substrain of Burkitt lymphoma Ramos cells [Ramos(-)] lacked GPI-anchored protein expression on the cell surface, which could be restored by transfecting the cells with *PIGA* cDNA^18^. Two other Burkitt lymphoma cell lines, Daudi and Akata, were also found to contain a large GPI-anchor-deficient population^19,20^. In these cells, transcriptional silencing of *PIGY* causes the lack of GPI-anchored proteins^19^. BLaER1 cells originate from the B cell precursor leukemia RCH-ACV cell line^5^. Although it has not been studied here, epigenetic silencing of the *PIGH* gene likely also occurs in the parental cell line. In general, GPI anchor deficiencies have so far only been described for cell lines derived from B cell lymphomas, suggesting a functional role in these cancer types. A recent study showed that adult B-lymphoblastic leukemia (ALL) patients frequently harbor GPI anchor-negative B cell populations^21^. This effect was found to originate from epigenetic silencing of *PIGH*, leading to a complete loss of its mRNA expression, similar to BLaER1 cells. As the RCH-ACV cell line was also derived from an ALL patient, a loss of PIGH expression might be a common feature for this type of B cell cancer. While a lack of GPI-anchored protein expression could contribute to cancer cell malignancy, it might also simply be a side effect induced by a high mutation rate. The latter hypothesis is supported by the finding that GPI anchor-deficient lymphocytes do not have survival or growth advantages in mice^22,23^. Further studies will be needed to provide a more comprehensive analysis of the role of GPI anchor loss in the development of B cell leukemia in humans.

## Methods

### Ethics statement

The studies involving human peripheral blood mononuclear cells (PBMCs) were approved by the local ethics committee (Ethikkommission der Medizinischen Fakultät Bonn) according to the ICH-GCP guidelines. Written informed consent was provided by voluntary blood donors.

### Primary cells and cell lines

Human PBMCs were isolated from whole human blood of healthy, voluntary donors by Ficoll-Hypaque density gradient centrifugation (Biochrom). PBMCs were cultured in RPMI-1640 medium supplemented with 10% FCS, 100 U/ml penicillin, and 100 µg/ml streptomycin. BLaER1 cells were kindly provided by Thomas Graf, Centre for Genomic Regulation, Barcelona, Spain. BLaER1 and THP-1 cells were cultivated in RPMI-1640 medium supplemented with 10% FCS, 100 U/ml penicillin, and 100 µg/ml streptomycin. HEK-Blue IFN-α/β cells, purchased from Invivogen, were cultured in DMEM supplemented with 10% FCS, 100 U/ml penicillin, and 100 µg/ml streptomycin.

Monoclonal BLaER1 cell lines were generated by seeding as single-cell clones via limited dilution and expansion: Per U-bottom 96-well plate, 25 cells were suspended in 10 ml medium and 100 µl were seeded per well (0.25 cells per well). After three days, wells containing cells were identified by microcopy. After two weeks of culture, clones were transferred to cell culture flasks and further expanded.

### Oligonucleotides

The RNA oligonucleotide 9.2s was ordered from Biomers. DNA oligonucleotides were ordered from Integrated DNA Technologies (IDT). Sequences are shown in Table 1.

**Table 1:** DNA and RNA oligonucleotide sequences.

Name	Sequence (shown 5′ to 3′)
**qRT-PCR primers**	
TLR4 fwd	CCCTGAGGCATTTAGGCAGCTA
TLR4 rev	AGGTAGAGAGGTGGCTTAGGCT
MD-2 fwd	CTGAAGGGAGAGACTGTGAATAC
MD-2 rev	GGCTCCCAGAAATAGCTTCA
ITGAM fwd	ACAACCCTAACCCAAGATCAC
ITGAM rev	AAACAGCTCTCGTACCACTTT
FCGR1B fwd	CCTTGAGGTGTCATGCGTG
FCGR1B rev	AAGGCTTTGCCATTTCGATAGT
CD14 fwd	GATTACATAAACTGTCAGAGGC
CD14 rev	TCCATGGTCGATAAGTCTTC
PIGH fwd1	GCCATCTTCATCACCCTCTTAG
PIGH rev1	CTGAATGCCAAGGGAATCAATG
PIGH fwd2	GCTGGACTGCTTGATTGAAGTAT
PIGH rev2	AAGACAATGCTGGCCTTCTG
PIGH fwd3	AGTCATGGTGTCAAGCACATTA
PIGH rev3	TCCAATTTCTGTCTGGCCTTT
PIGH fwd4	GCCATTTACATGCAGAAGGT
PIGH rev4	GACTGTGTCCACCTGATGGT
PIGH fwd5	GAGCAGGTGACTGGAAATCTAA
PIGH rev5	CCGAAAGTGTTCTTTGCTGAC
PIGH fwd6	GCTAAGCATGTTCAGGTTTACTTT
PIGH rev6	CCAGTACTGAAGGGCTTGTT
PIGH fwd7	GGAGGATGAGCGGAGCTTT
PIGH rev7	AGCGAACGCAGCGAGAG
PIGH fwd8	GGCGTCATGGAGGATGAG
PIGH rev8	CAGAATTCCCGGCAGGAC
PIGH fwd9	GGTGATTTACTACCTCTGCATCTT
PIGH rev9	AAGACGGGTACTACTTGGGATA
PIGH fwd10	TTCCGTACTCCAAACCATCAG
PIGH rev10	ATTTCCAGTCACCTGCTCTATG
PIGH fwd11	GGGAGATGACTCTTAAGCCATAG
PIGH rev11	GCTCTAAGATGCACTACATCCATAA
PIGH fwd12	GCAGGTGACTGGAAATCTAAC
PIGH rev12	CTCCTTTGGTAAAGTAGGCAAA
PIGH gDNA fwd	TACTGCGTTCAGCTGATTTAGG
PIGH gDNA rev	CCTCCAGAGAGGCCTGTAATA
**Cloning primers**	
PIGH GA fwd	GAATTTCGACCCGGATCCGCGGCCGCGCCACCATGGAGGATGAGC
PIGH/PIGA GA rev	CCCCTACCCGGTAGAATTCACGCGTTCACTTATCGTCGTCATCCTTGTAATC
PIGA GA fwd	GAATTTCGACCCGGATCCGCGGCCGCGCCACCATGGCCTGTAGAG
**RNA stimuli**	
3pRNA, sense	ppp-GGCCGAGACCUCGAAGAGAACUCU
3pRNA, antisense	ppp-AGAGUUCUCUUCGAGGUCUCGGCC
IVT4 template for in vitro transcription, sense	TTGTAATACGACTCACTATAGGGACGCTGACCCAGAAGA TCTACTAGAAATAGTAGATCTTCTGGGTCAGCGTCCC
IVT4 template for in vitro transcription, antisense	GGGACGCTGACCCAGAAGATCTACTATTTCTAGTAGATCT TCTGGGTCAGCGTCCCTATAGTGAGTCGTATTACAA
9.2s	AGCUUAACCUGUCCUUCAA
**DNA stimuli**	
G3-YSD, sense	GGGAAACTCCAGCAGGACCATTAGGG
G3-YSD, antisense	GGGTAATGGTCCTGCTGGAGTTTGGG

The sequences of DNA and RNA oligonucleotides used for qRT-PCR, cloning and cell stimulation are shown.

### Stimulatory nucleic acids

9.2s RNA was synthesized by Biomers. IVT4 was generated by in vitro transcription (IVT) with a commercial In vitro T7 Transcription Kit (Thermo Fisher Scientific) using annealed DNA oligonucleotides as a template (see Table 1), as described before^24^. 3pRNA was chemically synthesized as described before^25^. For hybridization of G3-YSD, DNA was heated in NEB2 buffer to 95 °C for 5 min and was then cooled to RT at a cooling rate of 1 °C/min. pDNA (pBluescript) was isolated from transformed *E. coli* K12 with a PureLink HiPure Plasmid Filter Midiprep Kit (Life Technologies).

### Plasmids

For lentiviral overexpression, human PIGH and PIGA with C-terminal Flag tags were subcloned into a pLVX-puro-EF1α vector (HIV-1 5′-LTR—HIV-1 packaging signal ψ—central polypurine tract—EF1α promotor—Open reading frame—PGK promotor—puromycin resistance—WHP posttrancriptional response element—HIV-1 3′-LTR—ampicillin resistance) via Gibson assembly.

### Transdifferentiation of BLaER1 cells

BLaER1 cells (3 × 10^5^ per well of a 24-well plate) were seeded in 900 µl medium containing 100 nM 17-estradiol (Sigma Aldrich), 10 ng/ml human IL-3 (Peprotech), and 10 ng/ml human M-CSF (Peprotech) and incubated for 5 days.

### Stimulation of cells

Cells were stimulated in duplicate with TLR ligands or oligonucleotides. Human PBMCs, transdifferentiated BLaER1 cells, or THP-1 cells (2 × 10^5^, 1.5 × 10^5^, or 6 × 10^4^ per well of a 96-well plate, respectively) were seeded in 150 µl RPMI-1640 supplemented with 10% FCS, 100 U/ml penicillin, and 100 µg/ml streptomycin. LPS-EK ultrapure, R848, TL8-506, CL264, or Pam3CSK4 (all from InvivoGen) were diluted in 50 µl medium per well and added to the cells. Final concentrations are indicated in the figures. RIG-I (3pRNA, IVT4) and cGAS agonists (pDNA, G3-YSD) were transfected with Lipofectamine 2000 according to the manufacturer’s instructions, giving final stimuli concentrations between 1 and 500 ng/ml, as indicated in the figures. Synthetic RNA ligand 9.2s was complexed with poly-L-Arginine of 5–15 kDa (pArg, Sigma-Aldrich, P4663) as delivery agent (1.8 µg per µg RNA). Per well of a 96-well plate, pArg and RNA were diluted in 25 µl PBS and incubated for 10 min before being added to the cells at a final concentration of 1 or 5 µg/ml RNA.

### Cytokine detection

Sixteen hours after stimulation, cell-free supernatant was collected and IL-6, TNFα, and IL-1β were measured by ELISA (BD Biosciences, 555220, 555212, 557953) according to the manufacturer’s instructions.

### HEK-Blue assay

HEK-Blue IFN-α/β reporter cells (InvivoGen) featuring type I IFN-inducible secreted embryonic alkaline phosphatase (SEAP) were seeded (6 × 10^4^ per well of a 96-well plate) in 180 µl DMEM supplemented with 10% FCS, 100 U/ml penicillin, and 100 µg/ml streptomycin. 20 µl of diluted or undiluted BLaER1 cell supernatant harvested 16 h after stimulation or 20 µl of medium containing IFN-α2a (Miltenyi Biotec, 130-093-874, standard ranging from 4000 to 62.5 U/ml) were added to HEK-Blue IFN-α/β cells. After incubation for 24 h at 37 °C, the supernatant was harvested and incubated with 1 volume 10 mg/ml p-nitrophenyl phosphate (pNPP, Sigma-Aldrich, 71768) in pNPP buffer (100 mM Tris, 100 mM NaCl, 5 mM MgCl_2_, pH 9.5). Absorption at 405 nm was measured to determine the formation of p-nitrophenol by SEAP activity.

### Flow cytometry

Differentiated or undifferentiated BLaER1 cells (3 × 10^5^) were washed with FACS buffer (2% FCS, 2 mM EDTA, PBS) once and incubated for 15 min in 25 µl Fc Receptor Blocking Solution (BioLegend, 422302, 1:100 in FACS buffer) at 4 °C. 25 µl of isotype control (BioLegend, 400220 or 400120, 1:50) or specific antibody for CD14, CD48, CD55, CD11b, CD45, CD64 (BioLegend, 301808, 336713, 311311, 301309, 368511, 305013, all 1:50), CD36, CD163, or CD191 (Miltenyi Biotec, 130-100-307, 130-100-612, 130-100-360, all 1:50) was added and incubated for 15 min at 4 °C. Cells were washed twice with FACS buffer and fixation was performed for 20 min in 4% paraformaldehyde/PBS at RT. After two further washings in FACS buffer, samples were measured on an LSRII (BD Biosciences) or Attune NxT (Thermo Fisher Scientific) flow cytometer.

Data analysis was performed using Flowjo (Treestar). After doublet exclusion, live cells were identified by GFP expression. The percentage of cells expressing the protein of interest (CD14, CD48, CD55, CD11b, CD45, CD64, CD36, CD163, or CD191) among GFP-positive cells was determined using matched isotype controls for gating.

### qRT-PCR

BLaER1 cells (1 × 10^6^) were lysed in 350 µl RLT buffer (Qiagen) and frozen at -80 °C for at least 5 min. After thawing, 1 volume of 70% ethanol was added and the sample was loaded onto a Zymo III column (Zymo). The column was washed sequentially with 1 volume RW1 buffer (Qiagen) and 1 volume RNA wash buffer (Zymo). The column was dried by centrifugation at maximum speed for 2 min and the RNA was eluted with RNase-free distilled H_2_O. Residual genomic DNA was digested by DNase I, and reverse transcription was performed with random hexamer primers and RevertAid Reverse Transcriptase (all from Thermo Fisher Scientific) according to the manufacturer’s instructions. For qRT-PCR, 5 × EvaGreen QPCR Mix II (ROX) (Bio-Budget) was used. The reaction was performed in a QuantStudio 5 Real-Time PCR cycler (Thermo Fisher Scientific). Primers were tested for efficiency using cDNA dilution.

### Immunoblotting

After washing once with PBS, cells were resuspended in RIPA lysis buffer supplemented with cOmplete Protease Inhibitor (Roche) and incubated on ice for 30 min. Lysates were cleared by centrifugation at 21,000× g for 10 min at 4 °C. The protein concentration was determined using a Pierce BCA Protein Assay Kit (Thermo Fisher Scientific) according to the manufacturer’s instructions and 10–25 µg of protein were loaded on an SDS-PAGE gel. Gel electrophoresis was performed for 90 min at 110 V. Proteins were blotted onto a nitrocellulose membrane for 90 min at 450 mA. CD14 and GFP were detected with primary antibodies D7A2T and 4B10 (Cell Signaling Technology, 1:1000), respectively. The housekeeping protein β-actin was stained with primary antibody 926-42212 (LI-COR Biosciences, 1:3000). PIGH and PIGA were detected with a Flag-tag primary antibody (Genscript, A00187, 1:1000). As secondary antibodies, goat anti-rabbit IRDye800CW, goat anti-mouse IRDye800CW, and goat anti-mouse IRDye680 (LI-COR Biosciences, 1:10,000) were used. Blots were recorded on an Odyssey FC Dual imaging system (LI-COR Biosciences).

### Determination of cell viability by MTT assay

Transdifferentiated BLaER1 cells (1.5 × 10^5^ per well of a 96-well plate) were seeded in 200 µl RPMI-1640 supplemented with 10% FCS, 100 U/ml penicillin, and 100 µg/ml streptomycin and incubated at 37 °C for 16 h. After carefully removing 150 µl of supernatant, 50 µl of MTT-containing medium were added to the cells to achieve a final MTT concentration of 1 mg/ml. Cells were incubated for 3 h at 37 °C, then the reaction was stopped by adding 100 µl of 10% SDS. After further incubation overnight at 37 °C, the absorption at 595 nm was measured.

### 3′ mRNA sequencing

Undifferentiated BLaER1 cells (1 × 10^6^) were resuspended in 500 µl TRIzol Reagent (Thermo Fisher Scientific) and their total RNA was purified according to the manufacturer’s instructions. Libraries of sequences close to the 3′ end of polyadenylated RNA were generated with a QuantSeq 3′ mRNA-Seq Library Prep Kit FWD (Lexogen) and sequenced on an Illumina HiSeq1500 device.

Sequencing reads were aligned to the human reference genome using STAR^26^. The transcripts were quantified with HTSeq^27^. Differential expression analysis was performed using edgeR^28^.

### Sanger sequencing of PIGH cDNA

After reverse transcription of RNA from undifferentiated BLaER1 cells, PIGH cDNA was amplified in a PCR reaction with gene-specific primers (PIGH fwd7, PIGH rev12) using 5 × EvaGreen QPCR Mix II (ROX) (Bio-Budget). Sanger sequencing of the PCR products from GPI-positive and GPI-negative monoclonal cell lines was performed by Microsynth Seqlab GmbH (Göttingen, Germany) using the PIGH fwd7 primer.

### Sanger sequencing of PIGH genomic DNA

BLaER1 cells (2 × 10^5^) were pelleted and resuspended in 40 µl PBND buffer (50 mM KCl, 10 mM Tris–HCl, 2.5 mM MgCl_2_, 0.1 mg/ml gelatin, 0.45% v/v IGEPAL, 0.45% v/v Tween-20) supplemented with Proteinase K. Genomic DNA (gDNA) was obtained by incubation at 55 °C for 60 min, followed by heat inactivation of the enzyme at 95 °C for 10 min. PIGH DNA was amplified in a DreamTaq PCR reaction according to the manufacturer’s instructions (Thermo Fisher Scientific) using gene-specific primers (PIGH gDNA fwd, PIGH gDNA rev). Sanger sequencing of PCR products from GPI-positive and GPI-negative monoclonal cell lines was performed by Microsynth Seqlab GmbH (Göttingen, Germany) using the PIGH gDNA fwd primer.

### Sequence analysis

Alignment of sequences obtained by Sanger sequencing to the Reference Sequence (RefSeq) and open reading frame (ORF) prediction were performed with ApE. Poly Peak Parser was used for peak parsing and deconvolution^29^.

### Treatment of cells

Undifferentiated BLaER1 cells (5 × 10^5^ per well of a 24-well plate) were seeded in 1 ml medium (untreated), or 1 ml medium containing 0.4 µM or 2 µM 5-aza-2′-deoxycytidine (Abcam, 2353-33-5). On days 2 and 4, cells were subcultured with fresh medium (with or without 5-aza-2′-deoxycytidine). CD48 and CD55 surface expression were assessed by flow cytometry 2, 4, and 7 days after the initial treatment.

### Lentiviral overexpression

Lentiviral vectors were produced by transfection of HEK 293FT cells with lentivector (1.6 µg of pLVX-puro-EF1α-PIGH-Flag, -PIGA-Flag or -GFP) and packaging plasmids (0.4 µg, 0.6 µg, and 1.1 µg of pRSV-rev, pMD2.G, and pMDL.g, respectively) using calcium phosphate transfection. Viral supernatants were harvested 72 h post transfection. BLaER1 cells were resuspended in virus supernatant and spin infection was performed (centrifugation at 600 × g for 60 min at 32 °C). The next day, the medium was replaced by Puromycin-containing medium (2.5 µg/ml) to select for cells containing the construct of interest. After 3 days, the medium was exchanged to Puromycin-free medium.

### Statistical analysis

Statistical analysis was performed in GraphPad Prism 7. To compare cells with high responsiveness to LPS (GPI-positive) to cells with lower responsiveness to LPS (GPI-negative), data from up to three monoclonal cell lines were pooled. As such pooled data cannot be assumed normally distributed, non-parametric Mann–Whitney tests were performed to compare both groups. Two-way ANOVA followed by Dunnett’s multiple comparisons test was used to statistically analyze differences between control cells and cells transduced with GFP, PIGH-Flag, or PIGA-Flag. Stimulation experiments with GFP-transduced and PIGH-Flag-transduced cells were statistically analyzed by performing unpaired t tests. * indicates a *p* value ≤ 0.05, ** a *p* value ≤ 0.01, *** a *p* value ≤ 0.001 and **** a *p* value ≤ 0.0001. Repetition and error bases are defined in the respective figure legends.

## Supplementary Information

Supplementary Information.

## Data Availability

3′ mRNA sequencing data generated in this study are uploaded at GEO (GSE169623).
