# Author Correction: An epigenetic GPI anchor defect impairs TLR4 signaling in the B cell transdifferentiation model for primary human monocytes BLaER1

**DOI:** 10.1038/s41598-021-97277-5

**Published:** 2021-08-30

**Authors:** Julia Wegner, Thomas Zillinger, Thais Marina Schlee-Guimaraes, Eva Bartok, Martin Schlee

**Affiliations:** 1 Institute of Clinical Chemistry and Clinical Pharmacology, University Hospital Bonn, Bonn, Germany; 2 Unit of Experimental Immunology, Department of Biomedical Sciences, Institute of Tropical Medicine, Antwerp, Belgium; 3 Present Address: Institute of Immunology, Philipps-University Marburg, Marburg, Germany

Correction to: *Scientific Reports* https://doi.org/10.1038/s41598-021-94386-z, published online 22 July 2021

The original version of this Article contained an error in the spelling of the author Thais Marina Schlee-Guimaraes which was incorrectly given as Thais Schlee-Guimaraes.

As a result, the Author Contributions section now reads:

“J.W. performed the experiments. J.W., E.B., and M.S. conceived the experiments and wrote the paper. M.S. secured funding. E.B. and T.Z. provided reagents, expertise and feedback. T.M.S.-G. analyzed 3′ mRNA-sequencing data. All authors revised the manuscript.”

The original Article has been corrected.

