# Supplementary material for: An epigenetic GPI anchor defect impairs TLR4 signaling in the B cell transdifferentiation model for primary human monocytes BLaER1
Source: Sci Rep. 2021 Jul 22;11:14983. doi: 10.1038/s41598-021-94386-z (PMC8298422; doi:10.1038/s41598-021-94386-z)
Supplement: Supplementary file 1 — Supplementary Information. [file 41598_2021_94386_MOESM1_ESM.pdf]

SUPPLEMENTARY INFORMATION

An epigenetic GPI anchor defect impairs TLR4 signaling in the B cell transdifferentiation model for primary human monocytes BLaER1

Julia Wegner, Thomas Zillinger, Thais Schlee-Guimaraes, Eva Bartok, Martin Schlee

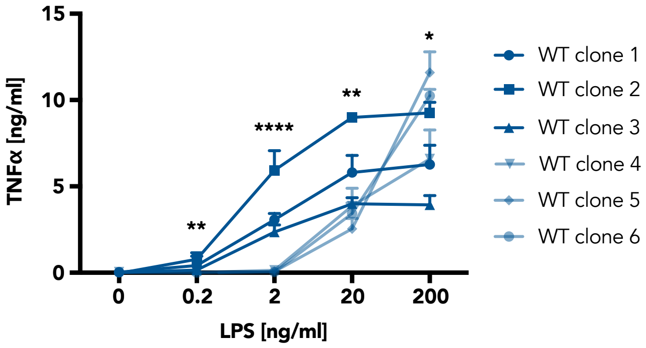

Figure S1. Differential LPS responsiveness of BLaER1 WT monoclonal cell lines

Transdifferentiated BLaER1 monoclonal cell lines were stimulated for 16 h with LPS, and TNFα in the supernatant was measured by ELISA. Values correspond to the mean + SD of n = 3 independent experiments. Mann-Whitney test was used to compare monoclonal cell lines with higher responsiveness to LPS (dark blue) to cell lines with lower responsiveness to LPS (light blue). Statistical significance is depicted as follows: * $p \le 0.05$, ** $p \le 0.01$, **** $p \le 0.0001$.

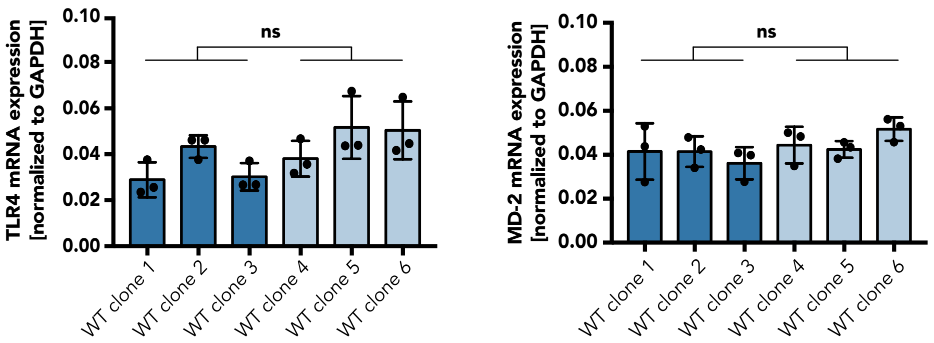

Figure S2. *TLR4* and *MD-2* mRNA expression are unchanged in clones with diminished LPS responsiveness

mRNA expression of *TLR4* and *MD-2* in transdifferentiated WT clones, measured by qRT-PCR. Columns correspond to the mean ± SD of n = 3 independent measurements, normalized to *GAPDH* mRNA expression. Individual values are shown as dots. Monoclonal cell lines with higher responsiveness to LPS (dark blue) were compared to cell lines with lower responsiveness to LPS (light blue) by Mann-Whitney test (ns – not significant).

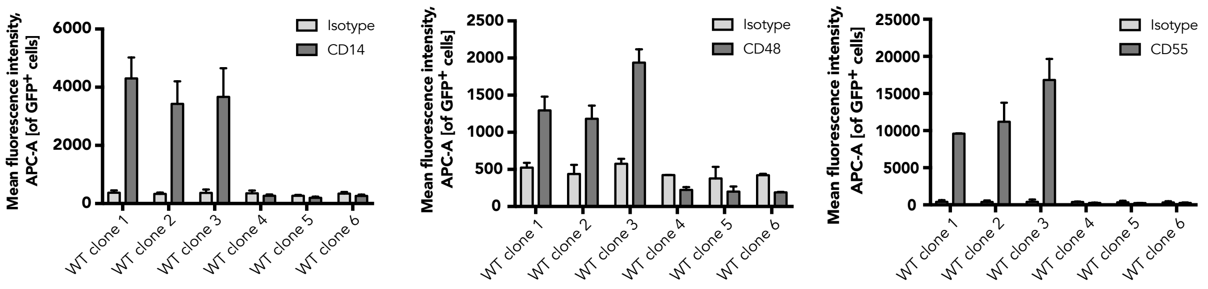

Figure S3. Loss of surface expression of GPI-anchored proteins

Surface expression of CD14, CD48, and CD55 of transdifferentiated GFP+ BLaER1 cells, assessed by FACS analysis. Shown is the mean + SD of n = 4 (for CD14) or n = 2 (for CD48 and CD55) independent measurements.

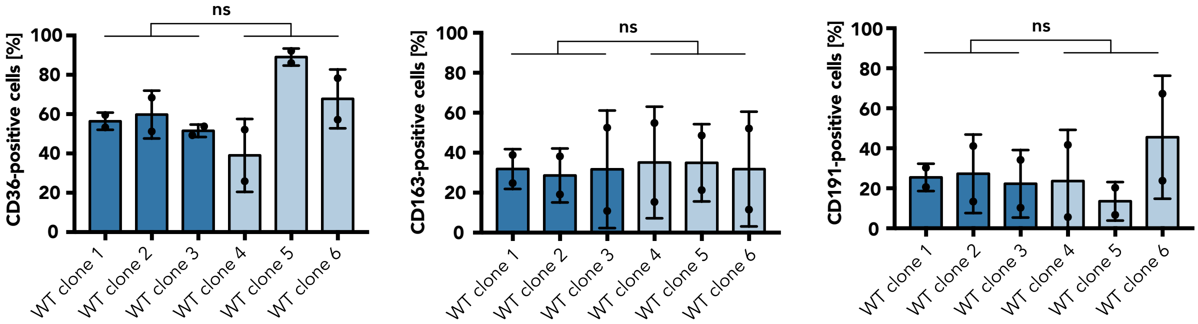

Figure S4. Unchanged surface expression of transmembrane proteins

Surface expression of the transmembrane proteins CD36, CD163, and CD191 in transdifferentiated BLaER1 clones was measured by flow cytometry. Shown is the percentage of cells expressing the protein of interest among all GFP-positive cells from n = 2 independent measurements (mean ± SD). Individual values are visualized as dots. Monoclonal cell lines with higher responsiveness to LPS (dark blue) were compared to cell lines with lower responsiveness to LPS (light blue) by Mann-Whitney test (ns – not significant).

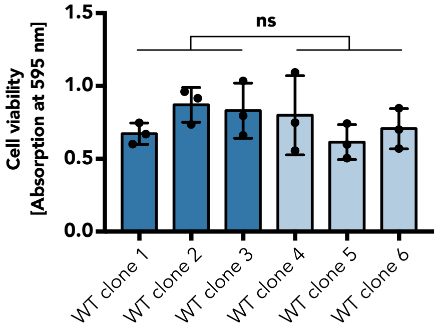

Figure S5. Cell viability is not affected by GPI anchor deficiency

Cell viability of transdifferentiated BLaER1 WT clones, assessed by MTT assay 16 h after seeding. Shown is the mean ± SD of n = 3 independent measurements. Individual values are depicted as dots. GPI-positive monoclonal cell lines with higher responsiveness to LPS (dark blue) were compared to GPI-deficient cell lines with lower responsiveness to LPS (light blue) by Mann-Whitney test (ns – not significant).

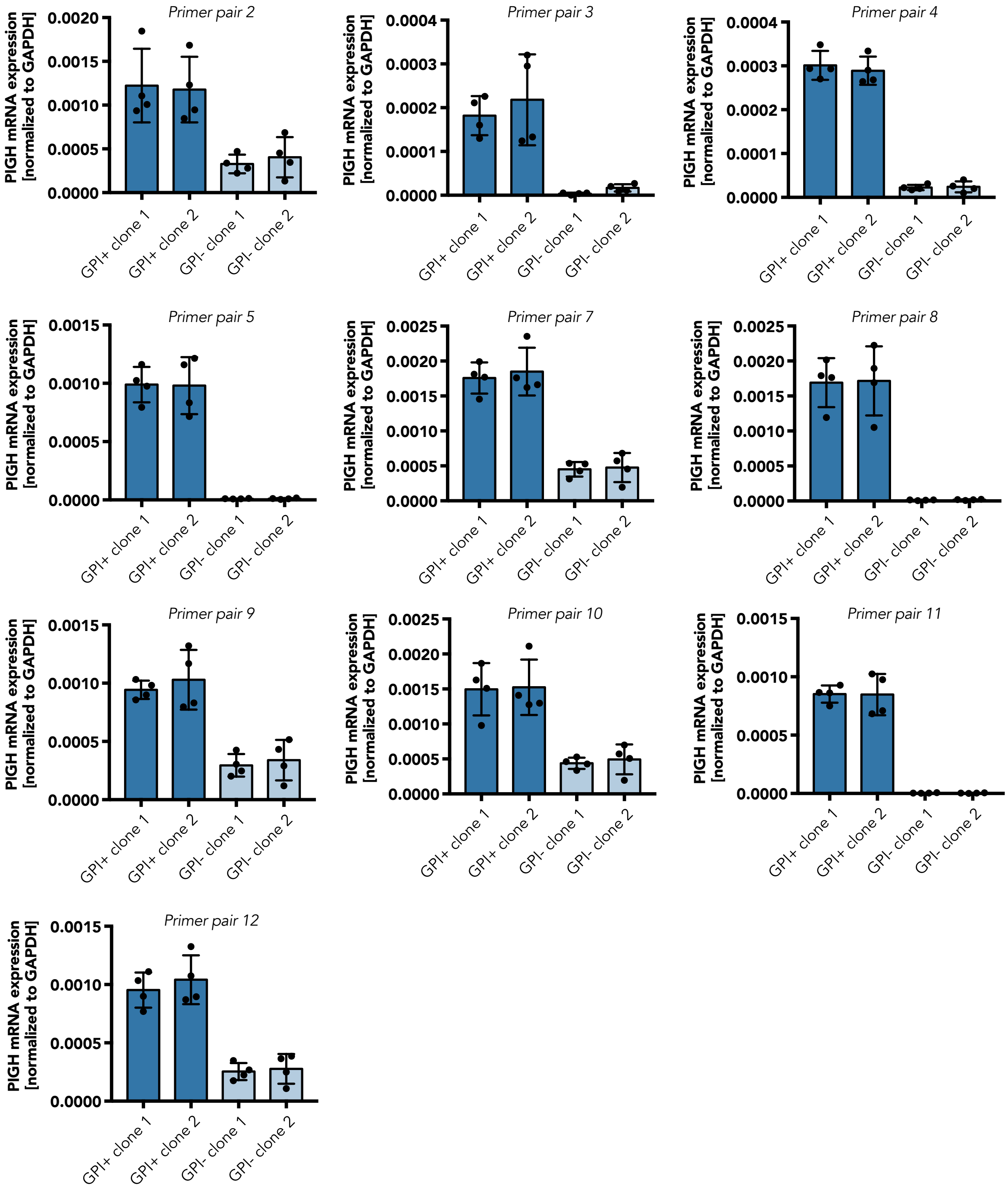

Figure S6. Primer-pair-dependent results of qRT-PCR for *PIGH* expression

PIGH mRNA expression of GPI-positive (GPI+) and -negative (GPI-) clones was quantified by qRT-PCR using different gene-specific primer pairs (2–5, 7–12) and normalized to *GAPDH* mRNA expression. Shown is the mean ± SD of data from n = 4 independent measurements. Individual values are visualized as dots.

a

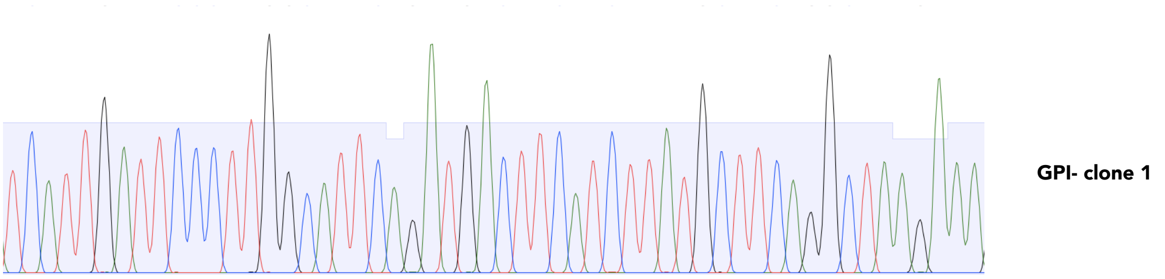

b

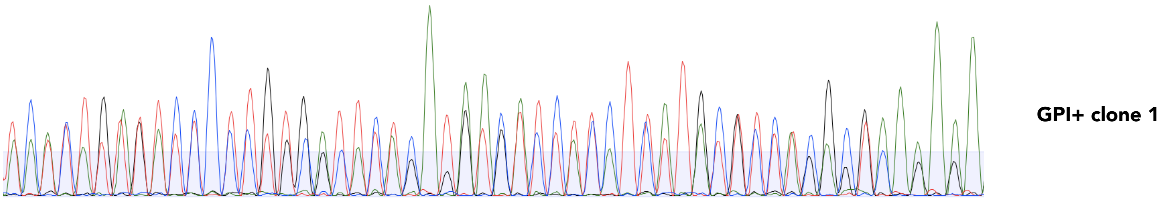

Figure S7. Sanger sequencing of *PIGH* cDNA reveals the presence of two different sequences in GPI-positive BLaER1 cells

After reverse transcription of mRNA from GPI-negative (GPI-) **(a)** and -positive (GPI+) **(b)** clones, *PIGH* cDNA was amplified using gene-specific primers and analyzed by Sanger sequencing. Overlapping peaks **(b)** indicate that two different *PIGH*-related sequences are present in the GPI+ clones.

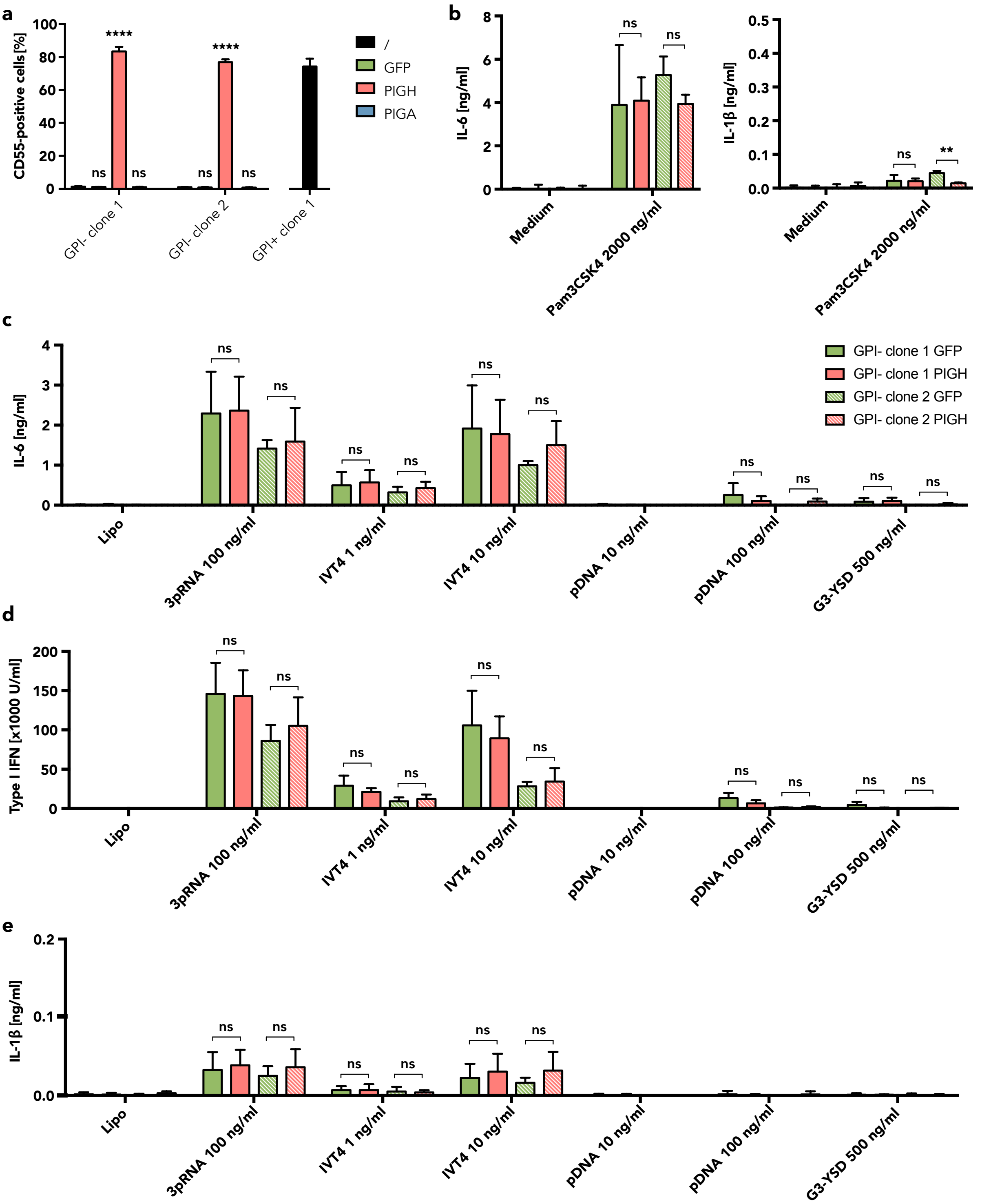

Figure S8. PIGH re-expression rescues CD55 surface expression but has no effect on TLR2/TLR1, RIG-I, and cGAS signaling

(a) GPI-negative BLaER1 clones that stably express GFP, PIGH-Flag, or PIGA-Flag were analyzed by flow cytometry. The percentage of CD55-expressing cells among GFP-positive cells is shown as the mean + SD from n = 3 independent measurements. The CD55 expression from a GPI-positive BLaER1 monoclonal cell line is shown on the right-hand side as positive control. Two-way ANOVA followed by Dunnett's multiple comparisons test was

used to determine statistical significance. Results from comparing cells transduced with either GFP, PIGH-Flag, or PIGA-Flag with the negative control (not transduced, /) are shown (ns – not significant, **** $p \leq 0.0001$).

(b) Concentrations of IL-6 and IL-1β in the supernatant were determined by ELISA 16 h after the stimulation of BLaER1 monoclonal cell lines stably expressing GFP or PIGH-Flag with the TLR2/1 ligand Pam3CSK4. Values correspond to the mean + SD of n = 3 independent experiments.

(c–e) BLaER1 clones stably expressing GFP or PIGH-Flag were stimulated for 16 h with RIG-I (3pRNA, IVT4) or cGAS (pDNA, G3-YSD) ligands and the supernatant was used to determine IL-6 **(c)** and IL-1β **(e)** concentrations by ELISA, or type I IFN concentration by HEK-Blue assay **(d)**. The mean + SD of n = 3 independent experiments is shown.

(b–e) BLaER1 cells stably expressing PIGH-Flag were compared to GFP-expressing control cells by performing unpaired t tests. Statistical significance is depicted as follows: ns – not significant, ** $p \leq 0.01$.

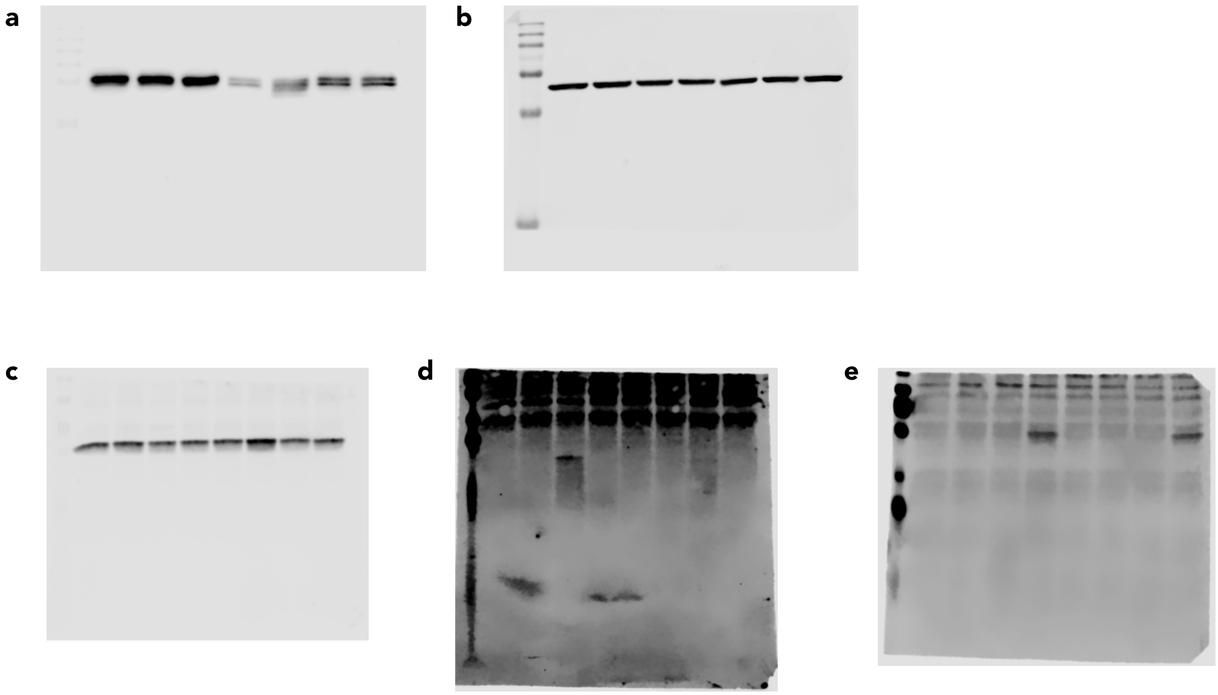

Figure S9. Full-length western blots

(a, b) Expression of CD14 **(a)** and housekeeping protein β-actin **(b)** in BLaER1 monoclonal cell lines with different LPS responsiveness. Full-length western blots from Figure 3b are shown.

(c–e) Expression of GFP, **(c)** PIGH-Flag **(d)** and PIGA-Flag **(e)** in BLaER1 monoclonal cell lines transduced with a lentivirus encoding for GFP, PIGH-Flag, or PIGA-Flag. Full-length western blots from Figure 6a are shown. Due to the low expression levels of PIGH and size differences between PIGH and PIGA, both proteins could not be detected on the same blot with the Flag antibody. Therefore, the same samples as for GFP and PIGA detection were run on a high-percentage acrylamide gel to visualize PIGH expression **(d)**.
